# The protective association of social cohesion on sex workers’ experiences of violence and access to community support: Impacts of resource sharing, trust and connection among a community-based cohort in Metro Vancouver, Canada (2010–2022)

**DOI:** 10.1371/journal.pone.0314749

**Published:** 2024-12-04

**Authors:** Jennie Pearson, Andrea Krüsi, Kate Shannon, Emma Ettinger, Deanna Kerrigan, Melissa Braschel, Charlie Zhou, Shira M. Goldenberg

**Affiliations:** 1 Interdisciplinary Studies Graduate Program, University of British Columbia, Vancouver, BC, Canada; 2 Division of Social Medicine, Department of Medicine, University of British Columbia, Vancouver, BC, Canada; 3 School of Criminology, Simon Fraser University, Burnaby, BC, Canada; 4 Department of Prevention and Community Health, Milken Institute School of Public Health, George Washington University, Washington, DC, United States of America; 5 Division of Epidemiology and Biostatistics, School of Public Health, San Diego State University, San Diego, CA, United States of America; Johns Hopkins University Bloomberg School of Public Health, UNITED STATES OF AMERICA

## Abstract

**Objectives:**

To measure recent social cohesion (resource sharing, trust and support) and its association with (1) sexual/physical violence, and (2) engagement with sex work-specific services among women sex workers in Metro Vancouver, Canada.

**Methods:**

Prospective data (January 2010-August 2022) were drawn from an open cohort of 900+ women sex workers. We developed multivariable logistic regression confounder models with generalized estimating equations (GEE) to examine associations between social cohesion and recent (1) physical/sexual violence and (2) engagement with sex work-specific services.

**Results:**

Of 918 participants, 36.8% were Indigenous and 32.1% were Black/Women of Colour. At baseline, the median social cohesion score was 19 (IQR 16–22), out of 36, with higher levels among participants who work with other sex workers. In separate multivariable confounder models with GEE, social cohesion was associated with lower odds of recent physical/sexual violence (Adjusted Odds Ratio 0.98 per point on scale, 95% Confidence Interval 0.97, 0.99) and recent use of sex work-specific services, although only statistically significant for physical/sexual violence.

**Conclusions:**

Findings support the need to eliminate policing of work environments that promote sex workers’ social cohesion and physical safety through full decriminalization.

## Introduction

Mutual aid—sharing of resources and tangible support done alongside demands for structural change- has historically played a key role in meeting everyday needs and long-term goals among marginalized and criminalized communities and those excluded from mainstream economies and labour protections [1], including sex workers [2]. Due to ongoing criminalization, state violence and stigma, mutual aid has particular necessity within sex worker organizing, and has remained essential to sex workers’ occupational health and safety (OHS), and wellbeing [3–5]. In the context of communal work environments (e.g., formal work venues, shared in-call spaces) or spatial proximity (common outdoor strolls), peer education, skill and resource sharing helps workers survive the gaps bad policy creates [6]. Within massage parlours, in clubs and on sets, sex workers help each other with shooting online content (e.g. photos, videos) and posting advertisements [5]. Spotting, providing condoms and sexual health education are everyday acts of peer support for outdoor and indoor workers [7, 8]. However, stigma and criminalization, including end-demand sex work laws, also hinder sex workers’ engagement with mutual aid (by limiting shared workspaces, use of condoms as evidence, censoring online resources, e.g.). In this cyclical manner, mutual aid among sex workers is based on meeting urgent and everyday needs, while advocating for law reform and systems-level change.

While ‘mutual aid’ is a broader, historical and political concept, *social cohesion* exists within mutual aid to specifically capture experiences and perceptions of resource sharing, trust and connectedness, predominantly among communities who work or live in the same setting [9, 10]. Indicators of social cohesion include: being able to rely on other sex workers for tangible (e.g., money or a place to stay) or emotional support, and that workers get along well with each other. In the global south, established best practices for advancing sex workers’ OHS include community empowerment programs that strengthen social cohesion [11]. Much of the existing research has focused on social cohesion among sex workers who share physical workspaces or participate in a local peer support program, with a focus on condom access and sexual health status [12–14]. Global research led by NSWP has expanded the use of social cohesion by identifying the potential of online spaces in supporting and reshaping sex workers’ social cohesion beyond physical work environments [15]. However, there remains opportunity to identify the impacts of social cohesion on sex workers’ diverse needs and priorities, and across diverse settings and criminalization models.

In the North American context, epidemiologic research assessing sex work social cohesion has largely focused on sexual health outcomes, finding high social cohesion linked with reduced client condom refusal, increased condom use for pregnancy prevention, and reduced odds of STI seropositivity [16–18]. In settings such as Canada, where sex workers and their work environments are extremely diverse, but also remain highly criminalized, there remains opportunity to identify a range of OHS benefits of social cohesion, and address what barriers remain. In 2014, the Canadian federal government implemented ‘end-demand’ criminalization (the Protection of Exploited Persons and Communities Act). These laws take aim at the demand for sex work—criminalizing clients and third party activities—and frame sex workers as victims of violence and exploitation, rather than as workers with rights and needs for occupational protections [19]. This end-demand approach has been adopted by an increasing number of global jurisdictions (e.g. Norway, Sweden, France) [20–22], and continues to be considered for implementation in additional settings, most recently in Scotland [23]. Despite popularity among lawmakers, end-demand approaches have been shown to have severe consequences for sex workers’ occupational conditions [22, 24] and perpetuate disproportionate policing and surveillance of sex work communities and their workspaces [25, 26]. Given the potential implications of the expansion of the end-demand regime, it is necessary to examine sex workers’ access to social cohesion within the context of end-demand, including settings such as Canada.

Therefore, aims of this study were to: 1. measure social cohesion (last 6 months), defined as perceived mutual aid, trust and support with other sex workers; and 2. identify the association between social cohesion (last 6 months) with a) sexual/physical violence, and b) engagement with sex work-specific services (e.g., drop-in spaces, harm reduction outreach) in the last 6 months among a large, diverse cohort of women sex workers in Metro Vancouver, Canada.

### Theoretical framework

This study recognizes sex work as not inherently violent or a health risk, but rather “a form of labour strongly shaped by intersecting structural factors” [27]. Our analysis is based on the understanding that sex work OHS is shaped by criminalization and policing, compounded by intersecting systems of oppression, and conditioned by work environments. By utilizing a structural determinants of health framework previously applied to sex workers’ OHS access [28], this analysis centres the diversity in lived experience and the interplay between structural factors at the macro, community, and work environment level. Macrostructural determinants include: social and economic systems governing sex work including punitive sex work laws [22]; immigration laws [29]; policing [30]; capitalism; colonialism and white supremacy [31]; patriarchy, transphobia and heteronormativism [32]; and stigma. Community level determinants include access to community-based outreach and services, and community participation (e.g., volunteering or peer work) [33]. Work environment level determinants include physical, social, policy, and economic features, such as type of work environment (indoor/outdoor), venue-based policies and collaboration between sex workers.

## Methods

### Data

This study draws on data from a community-based open prospective cohort, An Evaluation of Sex Workers Health Access (AESHA) which is based on community collaborations since 2005 and initiated recruitment in January 2010. Eligibility at baseline include identifying as a woman/femme while working, having exchanged sex for money in the last month, aged 14 and above, and able to provide informed consent. As previously described, our recruitment criteria are inclusive of diverse and fluid identities while capturing the ways that patriarchal gender norms shape participants’ experiences in sex work [27]. Eligibility is inclusive of cis and transgender women, transexual women and other transfeminine identities at enrolment.

As previously described [27, 33], time-location sampling was used to recruit participants through daytime and late-night (9 pm–2 am) outreach to outdoor sex work locations (e.g., streets, alleys) and indoor sex work venues (e.g., massage parlors, micro-brothels) across Metro Vancouver, BC. Online recruitment was also used to reach sex workers utilizing online solicitation spaces. Indoor and outdoor work environments are identified through community mapping and updated regularly by the outreach team. Since inception, current and former sex workers make up the research team, from research assistants and interviewers/outreach workers to coordinators. Participant recruitment completed on September 1, 2023. Further detail on AESHA’s origins is available in previous publications [34].

After obtaining informed consent, participants complete interviewer-administered questionnaires administered via REDCap [35] at baseline and semi-annual follow-up visits, eliciting responses on individual characteristics, work environments, structural factors, and health access and outcomes. Interviews are conducted at a study office in Vancouver or a confidential space of participants’ preference (e.g., home, work location). Currently, participants receive $80 Canadian at baseline and $65 at each follow-up for their time, expertise and travel. At each study visit, participants are offered voluntary HIV/STI/HCV testing and counseling by the sexual health research nurse and are offered referrals or STI treatment onsite, as needed and appropriate. Ethical approval for this study is provided by the Providence Health Care/University of British Columbia Research Ethics Board (REB number H09-02803).

### Study variables

#### Social cohesion

The main variable of interest was ‘social cohesion’, first assessed among sex workers in settings including India and Brazil, using a Social Cohesion Scale, a multi-item index that measures levels of perceived resource sharing, trust, solidarity and support among sex workers [10]. The scale has been previously adapted and validated with sex workers in Canada as part of AESHA, where a high level of internal consistency was indicated (Cronbach a = 0.919) [16, 17]. As described in previous AESHA publications [16], social cohesion among participants in the current study was based on a response to 12 items on a four-point scale ranging from strongly agree (3 points) to strongly disagree (0 points). Measures of social cohesion included items relating to being able to rely on other sex workers for money, advice, and a place to stay; social support when visiting a doctor; help with finding clients; help with violent perpetrators; and a sense that workers get along well with each other. The 12 item scores were summed to create a continuous measure for social cohesion, with a lowest possible score of 0, and a maximum possible score of 36. Social Cohesion was used as the outcome variable in Objective 1, and as the primary exposure of interest in Objective 2.

#### Occupational health and safety outcomes

For objective 2, time-updated OHS outcomes of interest included 1) sexual/physical violence and 2) engagement with sex work-specific services, in the last 6 months. Recent experiences of sexual/physical violence was defined as reporting any experiences of sexual or physical violence by any perpetrator (e.g., aggressors posing as clients, intimate partners, police), in the last six months. Recent engagement with sex work-specific services was defined by reporting engagement with or access of any sex work-specific services in Metro Vancouver (e.g., mobile outreach, drop-in spaces led by or tailored to sex workers providing harm reduction, peer support), in the last six months.

#### Other explanatory variables

Other explanatory variables of interest were selected *a priori* based on our theoretical framework, and literature related to sex work social cohesion and sex workers’ health and safety. All variables are derived from our study questionnaire and have been defined similarly in previous analyses [27, 33]. Individual-level variables included age (continuous), years in sex work (continuous), HIV seropositivity, and time-updated variables capturing experiences in the last six months, including any non-injection drug use (excluding alcohol and cannabis), any injection drug use, as well as good self-rated health. Macrostructural variables included racialization and Indigenous identity to examine the effects of colonization and racism, defined as Indigenous (inclusive of First Nations, Métis, or Inuit), Black/Women of Colour (e.g., East Asian, Southeast Asian) vs. white. Given the low proportion of participants who identified as Black in our sample (which is consistent with the Black population of BC (<2%)), we combined Black and Women of Colour to examine effects of racism among this group. We also considered mental health diagnoses, high school attainment, im/migration to Canada, sexual minority identity (gay, lesbian, bisexual, asexual, queer, other vs. heterosexual), and gender identity (cis women vs trans women, including, transgender women, transexual women and other transfeminine identities). Other structural variables included lifetime experiences of incarceration. All remaining structural variables captured experiences in the last 6 months, including: any police harassment while working, any unstable housing (i.e., living in an SRO, supportive housing, etc. vs. living in own apartment/house), and any barriers to healthcare.

Community and work environment level variables included primary place servicing clients in the last six months (work environment level), including outdoor/public (e.g., street, public washroom, car, tent), and informal (e.g., own or client’s place of residence) or formal indoor spaces (e.g., massage/beauty parlour, micro-brothel). This variable included a category for no recent sex work, as not all participants actively do sex work at every semi-annual study visit. Other work environment-level variables included having worked with other sex workers as a safety strategy.

#### Statistical analyses

Baseline descriptive statistics for independent variables were calculated as frequencies and proportions for categorical variables and measures of central tendencies (i.e., mean, median, interquartile range (IQR)) for continuous variables.

We next conducted bivariate analysis using linear regression with generalized estimating equations (GEE) and an exchangeable correlation matrix [36] to examine associations between social cohesion and individual and structural variables of interest over the study period. GEE was used to account for repeated measurements over time among the same participants. We subsequently developed two separate multivariable confounder models using logistic regression with GEE to identify the independent association between social cohesion and outcomes of (1) sexual/physical violence, and (2) engagement with sex work-specific services. We used GEE to address the clustered nature of repeated observations among the same participants across study visits. Correlations due to repeated measure was handled through a correlation matrix. Hypothesized confounders that were included in the full model included age, sexual minority at any study visit, racialization, primary place servicing clients, recent unstable housing, recent inconsistent condom use with clients, recent police harassment and recent non-injection drug use. To account for the noncollapsibility effect when using logistic regression [37], we also conducted a sensitivity analysis using manual backward selection on confounders. The equation and code underlying the GEE analysis can be found in Appendix 1 [S1 File]. Analyses were performed in SAS version 9.4 (SAS, Cary, NC), 95% confidence intervals are presented, and all p-values are two-sided. Missing data were handled using a complete case approach.

## Results

Analyses included 4179 observations collected amongst 918 participants from January 2010 –August 2022. Among participants, 338 (36.8%) were Indigenous and 295 (32.1%) identified as Black/Women of Colour. At baseline, 38.1% (n = 350) serviced clients in outdoor spaces, while 27.1%, (n = 249) serviced clients in informal indoor spaces (e.g., apartments), and 32.6% (n = 299) in formal in-call spaces. 35.7% of participants reported having experienced any recent physical/sexual violence. As well, a majority of participants (57.6%) recently engaged with sex work-specific services (e.g., mobile outreach, drop-in spaces led by or tailored to sex workers providing harm reduction, peer support). At baseline, the median social cohesion score was 19 (IQR 16–22), out of a possible 36 (Table 1).

**Table 1:** Baseline characteristics of women sex workers in Metro Vancouver who completed the social cohesion scale, Vancouver, Canada, 2010–2022 (N = 918).

Characteristic	Yes n (%)		No n (%)
***Individual and Interpersonal Factors***			
Social Cohesion (median, IQR)		19 (16–22)	
Age (median, IQR)		35 (28–43)	
Years working in sex work (median, IQR)		9 (3–18)	
Non-injection drug use^†^		593 (64.6)	323 (35.2)
Injection drug use^†^		365 (39.8)	551 (60.0)
Good self-rated health^†^		637 (69.4)	280 (30.5)
HIV Seropositivity		82 (8.9)	789 (86.0)
***Macrostructural level***			
*Racialization*			
Indigenous		338 (36.8)	
Black/Women of Colour		295 (32.1)	
White		285 (31.1)	
Im/migrated to Canada		281 (30.6)	637 (69.4)
Sexual Minority*		352 (38.3)	560 (61.0)
Gender Minority**		77 (8.4)	838 (91.3)
High school attainment		505 (55.0)	413 (45.0)
Any unstable housing^†^		656 (71.4)	259 (28.2)
Any barriers to healthcare^†^		600 (65.4)	316 (34.4)
Police harassment while working^†^		271 (29.5)	645 (70.3)
Any physical/sexual violence by any perpetrator^†^		328 (35.7)	537 (58.5)
***Community and Work Environment level***			
Engaged with sex work-specific services^†^		529 (57.6)	388 (42.3)
*Primary place servicing clients* ^†^			
Outdoor or public space		350 (38.1)	
Informal Indoor		249 (27.1)	
Formal Indoor		299 (32.6)	
Worked together with other sex workers as a safety strategy^†^		374 (40.7)	544 (59.3)

*Gay, lesbian, bisexual, asexual, queer

** Trans women inclusive of transgender women, transexual women and other transfeminine identities vs cisgender women

† In the last 6 months

In bivariable GEE analysis, increased social cohesion was associated with formal indoor work environments (3.43 per point increase on scale, 95% Confidence Interval (CI) 2.82, 4.03), good self-rated health (0.74 per point increase on scale, 95% CI 0.41, 1.07), and working with other sex workers as a safety strategy (1.89 per point increase on scale, 95% CI 1.56, 2.22) (Table 2).

**Table 2:** Bivariate GEE analysis of individual and interpersonal, macrostructural, community and work environment factors with social cohesion among women sex workers in Vancouver, Canada, 2010–2022 (N = 918).

	Unadjusted		
Characteristic	Co-Efficient	95%Confidence Interval	p—value
***Individual and Interpersonal Factors***			
Age	-0.05	(-0.08,-0.02)	0.001
Years working in sex work	-0.12	(-0.15,-0.09)	<0.001
Non-injection drug use^†^	-0.91	(-1.35,-0.48)	<0.001
Injection drug use^†^	-0.92	(-1.41,-0.44)	<0.001
Good self-rated health^†^	0.74	(0.41, 1.07)	<0.001
HIV Seropositivity	-1.74	(-2.72, - 0.75)	<0.001
***Macrostructural***			
*Racialization*			
White	REF		
Indigenous	0.08	(-0.71, 0.87)	0.844
Women of colour	3.31	(2.52, 4.10)	<0.001
Im/migrated to Canada	3.26	(2.65, 3.88)	<0.001
Sexual Minority*	-0.31	(-0.99, 0.36)	0.363
Gender Minority**	-0.09	(-1.27,1.01)	0.880
High school attainment	1.85	(1.22, 2.48)	<0.001
Any unstable housing^†^	-1.19	(-1.65, -0.73)	<0.001
Any barriers to healthcare^†^	-0.06	(-0.37, 0.25)	0.716
Police harassment while working^†^	0.63	(0.10, 1.17)	0.021
Any physical/sexual violence by any perpetrator^†^	-0.41	(-0.75, -0.08)	0.016
***Community and Work Environment Level***			
Engaged with sex work-specific services^†^	-0.69	(-1.07, -0.31)	<0.001
*Primary place servicing clients* ^†^			
Outdoor or public space	REF		
Informal Indoor	0.50	(0.09, 0.91)	0.017
Formal Indoor	3.43	(2.82, 4.03)	<0.001
Worked together with other sex workers as a safety strategy^†^	1.89	(1.56, 2.22)	<0.001

*Gay, lesbian, bisexual, asexual, queer

** Trans women inclusive of transgender women, transexual women and other transfeminine identities vs cisgender women

† In the last 6 months

In separate multivariable GEE confounder models (Table 3) adjusted for key confounders, increased social cohesion was associated with lower odds of recent physical/sexual violence (Adjusted Odds Ratio (aOR) 0.98 per point on scale; 95% CI 0.97, 1.00; p = 0.025) and recent use of sex work-specific services (aOR 1.00 per point on scale, 95% CI 0.98, 1.01; p = 0.829).

**Table 3:** Relationship between social cohesion and recent physical/sexual violence and use of sex work-specific services among sex workers in Vancouver, BC, [2010–2022] (n = 918).

Outcome	Exposure: Social Cohesion	
	Unadjusted Odds Ratios (95% CI)	Adjusted Odds Ratios (95% CI)
***Recent physical/sexual violence*** ^**†**^	-0.98 (0.97, 0.99)	**0.98 (0.97, 0.99)** *
***Recently engaged with sex work specific services*** ^***†***^	-0.98 (0.96, 0.99)	**1.00 (0.98, 1.01)** **

* Adjusted for hypothesized confounders of age, unstable housing, primary place servicing clients, recent police harassment, recent inconsistent condom use with clients and recent non-injection drug use.

**Adjusted for hypothesized confounders of racialization, unstable housing, primary place servicing clients, recent police harassment and recent non-injection drug use

^†^ In the last 6 months

## Discussion

This study addressed social cohesion–perceptions of resource sharing, trust and support–over twelve years in a cohort of sex workers across diverse work environments. Among our sample of sex workers based in Metro Vancouver, increased social cohesion was associated with lower odds of recent physical/sexual violence and increased odds of recently using sex work-specific services, although this was only statistically significant for recent physical/sexual violence. Overall, we found mid to high levels of social cohesion, consistent with findings across global settings, while revealing increased social cohesion among sex workers who work in formal indoor work environments, and who work with others as a safety strategy.

When measuring social cohesion among a diverse cohort of women sex workers, our study found high levels of social cohesion among those who service clients in formal, indoor venues (e.g. massage parlours), as well as among im/migrant sex workers, who are more likely to work within such spaces [38]. Previous research has found that im/migrant sex workers face unique barriers to more formalized community engagement models and sex work-specific services due to compounding criminalization, language barriers, and exacerbated stigma [33, 39]. However, our research suggests that working in communal spaces and in collaboration with peers facilitates social cohesion and can support trust-building among sex workers. At the same time, a plethora of research has demonstrated the high levels of racialized policing faced by formal indoor venues [38, 39], which limit sex workers’ access to sex work- specific services as well as general health services [40, 41]. Recent research with sex workers and third parties in Metro Vancouver has found that peer support within collaborative work environments is necessary for filling gaps related to sexual health education, harm reduction, and support for sex workers who experience violence [8]. While our current study demonstrates the benefits of collaborative workspaces in promoting social cohesion, there remains an urgent need to address the policies that create these support gaps.

Additionally, when measuring social cohesion levels among sex workers, we found high scores among participants who reported working with other sex workers as a safety strategy. Working in isolation, indoors and outdoors, has been shown to increase sex workers’ risk of violence [42–44], while leaning on other sex workers who act as spotters, or knowing that other workers are nearby, has helped sex workers exert more agency and negotiate boundaries [7, 45]. Despite the OHS benefits of collaborative workspaces, criminalization continues to isolate sex workers across diverse work environments. Previous Vancouver-based research has found outdoor sex work spaces face high levels of police surveillance due to the overlapping criminalization of sex work, drug use and poverty, forcing sex workers to work in secluded areas and away from other workers and sex work-specific services [42, 43, 46]. In the context of Vancouver, precarious or low-income workers, unhoused communities, and people living in single-room occupancy buildings, including many sex workers, are also being severely impacted by an ongoing housing crisis and city-enforced evictions and destruction of encampments [47, 48]. All of which impact sex workers’ access to collaborative and/or supportive outdoor and indoor work environments. Additionally, end-demand sex work laws criminalize collaborative indoor workspaces, and perpetuate fear of criminalization for sex workers who offer peer support. Our study builds on existing research and community knowledge by demonstrating the potential of collaborative work environments in promoting social cohesion and associated OHS benefits, including safety and access to services.

Lastly, we found that social cohesion was associated with lower odds of recent physical or sexual violence, echoing previous research in the global south which has shown that social cohesion among sex workers plays a critical role in supporting safety [10, 14]. For example, in Jamacia, where sex work is partially criminalised yet highly policed, social cohesion has been linked to reduced odds of violence and harassment experienced by sex workers, including client and intimate partner violence, as well as police violence [14]. Our study builds on this body of work by providing some of the first quantitative data on associations of social cohesion and violence within the North American context, and is the first to explore role of social cohesion in shaping sex workers’ experiences of violence under end-demand laws. While bivariate analysis demonstrated that sex workers reporting higher social cohesion faced increased odds of using sex worker-specific services, this effect did not retain statistical significance after adjustment for confounding. Our descriptive and bivariate findings build on community knowledge that when sex workers are able to work collaboratively, they are more likely to receive support and service referrals, engage in advocacy, and are better connected to the broader community [4, 5]. All of these have been linked to improved safety and wellbeing [7, 33, 49].

Ongoing barriers to sex worker social cohesion and collectivization in the context of Canada’s end-demand laws point to the need for full decriminalization of sex work, as recommended by sex worker-led organizations in Canada and globally [24, 50]. In a global review of sex work criminalization models, repressive policing of sex workers under partial or end-demand criminalization was associated with working in isolation and reduced OHS outcomes [22]. Whereas in decriminalized settings such as New Zealand, sex workers have experienced a reduced threat of arrest and harmful police interactions [51], and increased access to supportive workspaces [52]. By demonstrating the limitations of social cohesion under end-demand, our findings bring further attention to the ongoing call for full decriminalization, and an end to police surveillance of sex workers and their work environments, as a necessary first step in promoting social cohesion and ensuring sex workers have equal access to safe, collaborative work environments and tailored services.

### Strengths and limitations

Strengths of this study include its prospective nature, strong community collaborations, a large, diverse sample, and use of a validated and adapted scale as our primary outcome variable. Our framing and literature review draws heavily on literature and knowledge produced by current and former sex workers. Still, several limitations remain. This research cannot infer causality, and all data are self-reported. As with most research involving stigmatised populations, there is potential for social desirability and recall bias. However, our community-based and experiential team, training in non-stigmatising interview techniques are designed to mitigate this. The weekly time-location outreach conducted by our interview team, and frequent check-ins with participants help minimize loss to follow up. Time–location sampling also helped ensure a representative sample and minimize selection bias, however, findings may not be fully generalizable to all sex worker populations, including Black and Latinx sex workers, and those with diverse gender identities including men, masc and non binary sex workers. Further research is needed to explore the role of gender identify in shaping social cohesion, and which engages more diverse groups of women of colour sex workers, to address the impacts of diverse forms of racism on sex workers’ social cohesion. Finally, this analysis used GEE to model the clustered nature of the data (repeated measures across the same participants over time); we conducted sensitivity analyses using GLMM, which yielded similar results.

## Conclusions and implications for policy change

In summary, we identified an association between social cohesion and (a) reduced odds of physical/sexual violence, and (b) increased odds of engagement with sex work-specific services over 12 years in a diverse cohort of sex workers in Vancouver, Canada. However, this effect was only shown to be statistically significant after adjustment for confounding for the physical/sexual violence outcome. Our study is among the first to demonstrate outcomes linked to social cohesion beyond condom negotiation and sexual health status, as well as situate social cohesion within the broader legacy of mutual aid among sex work communities, and provide critical data on the relationship between social cohesion and sex workers’ OHS in a North American and end-demand context. Our results highlight the role of collaboration in supporting social cohesion, as well as sex workers’ collective strength and ability to care for one another. As well, by more broadly exploring social cohesion as a primary outcome variable, we were able to identify work environments and strategies that support higher levels of social cohesion, including formal indoor work environments and working with other sex workers as a safety strategy. This research supports the need to scale up sex work co-working spaces, in addition to safe and dignified housing options, as well as increase access to digital technologies that allow for greater communication and sharing of resources among sex workers. Lastly, the current research demonstrates a critical need to eliminate policing and state surveillance of sex work, through full decriminalization, including sex worker collectivization and indoor and outdoor workspaces, to better promote sex workers’ social cohesion, safety and access to tailored, sex worker-led services.

## Supporting information

S1 File Appendix 1. GEE analysis equation and code.
(DOCX)

## List of abbreviations

STI-: Sexually Transmitted Infection
HCV: Hepatitis C virus
OHS: Occupational Health and Safety
AESHA-: An Evaluation of Sex Workers’ Health Access
DTES: Downtown Eastside
