# Supplementary material for: The protective association of social cohesion on sex workers’ experiences of violence and access to community support: Impacts of resource sharing, trust and connection among a community-based cohort in Metro Vancouver, Canada (2010–2022)
Source: PLoS One. 2024 Dec 4;19(12):e0314749. doi: 10.1371/journal.pone.0314749 (PMC11616884; doi:10.1371/journal.pone.0314749)
Supplement: S1 File — GEE analysis equation and code. (DOCX) [file pone.0314749.s001.docx]

**Appendix 1. GEE Analysis Equation and Code**

When using recent physical/sexual violence as our outcome, the GEE model equation is:

Logit(P(Recent physical/sexual violence=Yes)) = \beta_{0} + \beta_{1}*Social Cohesion + + \beta_{2}*age + + \beta_{3}*unstable housing ++ \beta_{4}* primary place servicing clients + + \beta_{5}*recent police harassment + + \beta_{6}*recent inconsistent condom use with clients + + \beta_{7}*recent non-injection drug use

When using recent engagement with sex work-specific services as our outcome, the GEE model equation is:

Logit(P(Recently engaged with sex work specific services =Yes)) = \beta_{0} + \beta_{1}*Social Cohesion + + \beta_{2}*age + + \beta_{3}*unstable housing ++ \beta_{4}* primary place servicing clients + + \beta_{5}*recent police harassment + + \beta_{6}*recent inconsistent condom use with clients + + \beta_{7}*recent non-injection drug use”

Multivariate cofounder analysis in SAS:

%include "data location\data.sas";

ods path(prepend) work.templat(update);

proc template;

define style mystyles;

parent = styles.rtf;

style body from document /

marginbottom = 1in

margintop = 1in

marginleft = 1in

marginright = 1in;

end;

run;

%macro gee_confound(mydataset=, myoutfile=, myname=, myfull=, myinterest_ref=, mycatvar_ref=,

myoutcome=, myinterest=, myconfounders=, mysubject=);

proc genmod data=&mydataset descending;

class &mysubject &myinterest_ref &mycatvar_ref / param=reference;

model &myoutcome=&myinterest &myconfounders / dist=bin link=logit;

repeated subject=&mysubject/ type=EXCH;

ods select NObs GEEModInfo ConvergenceStatus GEEExchCorr GEEEmpPEst;

ods output GEEEmpPEst=temp;

run;

data temp;

set temp;

if parm="&myinterest";

*parameter and estimate of interest;

length var $80.;

var=&myname;

est=estimate;

full=&myfull;

keep var est full;

run;

%if &myfull=1 %then %do;

data &myoutfile;

set temp;

run;

%end;

%if &myfull=0 %then %do;

data &myoutfile;

set &myoutfile temp;

run;

%end;

quit;

%mend;

*Multivariate GEE macro;

proc template;

define table OddsRatioEst;

column effect est lcl ucl;

*column effect est lcl ucl probZ;

define header myheader;

text "Odds Ratio Estimates";

end;

define column effect;

header="Effect";

end;

define column est;

header="Point Estimate";

end;

define column lcl;

header="95% Lower Confidence Limit";

width=19;

end;

define column ucl;

header="95% Upper Confidence Limit";

width=18;

end;

end;

run;

%macro mvgee(mydataset=, myoutcome=, myvars=, myrefvars=, mysubject=, mycorr=);

data _null_;

set &mydataset;

run;

proc genmod data=&mydataset descending;

class &mysubject &myrefvars / param=ref;

model &myoutcome=&myvars/ dist=bin link=logit type3;

repeated subject=&mysubject/ type=&mycorr;

ods output GEEEmpPEst=beta;

ods exclude ParmInfo ClassLevels;

run;

data odds;

set beta;

length effect $70.;

if parm="Intercept" then delete;

effect=parm;

est=exp(estimate);

lcl=exp(lowerCL);

ucl=exp(upperCL);

format est lcl ucl 8.3;

run;

data _null_;

set odds;

file print ods=(template="OddsRatioEst");

put _ods_;

run;

quit;

%mend;

%macro mvgee_cont(mydataset=, myoutcome=, myvars=, mysubject=, mycorr=);

data _null_;

set &mydataset;

run;

proc genmod data=&mydataset descending;

class &mysubject / param=ref;

model &myoutcome=&myvars/ dist=bin link=logit type3;

repeated subject=&mysubject/ type=&mycorr;

ods output GEEEmpPEst=beta;

ods exclude ParmInfo ClassLevels;

run;

data odds;

set beta;

length effect $70.;

if parm="Intercept" then delete;

effect=parm;

est=exp(estimate);

lcl=exp(lowerCL);

ucl=exp(upperCL);

format est lcl ucl 8.3;

run;

data _null_;

set odds;

file print ods=(template="OddsRatioEst");

put _ods_;

run;

quit;

%mend;

/*** FULL SAMPLE ***/

/* OUTCOME 1: any_violence_l6m */

data master_mv_violence;

set master;

if any_violence_l6m=. or social_cohes_score=. or age=. or lgbq2s_any=. or race=. or housing_l6m=. or

serve_where_r=. or police_harass_l6m=. or unsafesex_any_l6m=. or ninj_l6m=. then delete;

run;*4179 to 3794;

*Check for multicollinearity;

ods graphics off;

proc reg data=master_mv_violence;

model any_violence_l6m = social_cohes_score age lgbq2s_any race housing_l6m

serve_where_r police_harass_l6m unsafesex_any_l6m ninj_l6m / vif;

run;

*VIF = 1.72;

/* OUTCOME 2: sexwork_outreach_l6m */

data master_mv_outreach;

set master;

if sexwork_outreach_l6m=. or social_cohes_score=. or age=. or lgbq2s_any=. or race=. or housing_l6m=. or

serve_where_r=. or police_harass_l6m=. or unsafesex_any_l6m=. or ninj_l6m=. then delete;

run;*4179 to 3994;

*Check for multicollinearity;

ods graphics off;

proc reg data=master_mv_outreach;

model sexwork_outreach_l6m = social_cohes_score age lgbq2s_any race housing_l6m

serve_where_r police_harass_l6m unsafesex_any_l6m ninj_l6m / vif;

run;

*VIF = 1.7;

/* GENERATING ADMINISTRATIVE DATA */

data admindata;

input field &:$20. desc &:$1500.;

datalines;

Project Name: Social cohesion on sex workersí experiences of violence and access to tailored services

Primary Name: Jennie Pearson

(Co) PI Name: Kathleen Deering

Analyst Name: Charlie Zhou

Request Name: Multivariate anlysis

Request Desc: This is the MV confounder Model, which using CURRENT model select method.

;

run;

%let outfile = "result location\result.doc";

ods rtf file = &outfile style = mystyles bodytitle startpage = never contents toc_data keepn;

* ADMINISTRATIVE INFORMATION;

ods rtf startpage = now;

ods proclabel = "1.0: ADMINISTRATIVE INFORMATION";

title "ADMINISTRATIVE INFORMATION";

proc report data = admindata noheader

style(report) = {rules = none frame = void}

style(column) = {font = (Courier, 10pt) just = l};

col field desc;

define field / style(column) = {cellwidth = 1.2in};

run;

* Frequencies of digital access and mutual aid responses;

ods rtf startpage = now;

ods proclabel = "2.0: Full Model, outcome is any_violence_l6m";

title1 j=left font='Arial' color=black bold "Full Confounder Model";

%mvgee(mydataset=master_mv_violence, myoutcome=any_violence_l6m, myvars=social_cohes_score age lgbq2s_any

race housing_l6m serve_where_r police_harass_l6m unsafesex_any_l6m ninj_l6m, myrefvars=lgbq2s_any(ref='0: No')

race(ref='0: White') housing_l6m(ref='0: No') serve_where_r(ref='1: Outdoor/public space') police_harass_l6m(ref='0: No')

unsafesex_any_l6m(ref='0: No') ninj_l6m(ref='0: No'), mysubject=aesha_code, mycorr=exch);

ods rtf startpage = now;

ods proclabel = "3.0: Most Parsimonious Confounder Model, outcome is any_violence_l6m";

title1 j=left font='Arial' color=black bold "Most Parsimonious Confounder Model";

%mvgee(mydataset=master_mv_violence, myoutcome=any_violence_l6m, myvars=social_cohes_score age

housing_l6m serve_where_r police_harass_l6m unsafesex_any_l6m ninj_l6m, myrefvars=lgbq2s_any(ref='0: No')

race(ref='0: White') housing_l6m(ref='0: No') serve_where_r(ref='1: Outdoor/public space') police_harass_l6m(ref='0: No')

unsafesex_any_l6m(ref='0: No') ninj_l6m(ref='0: No'), mysubject=aesha_code, mycorr=exch);

ods rtf startpage = now;

ods proclabel = "4.0: Full Model, outcome is sexwork_outreach_l6m";

title1 j=left font='Arial' color=black bold "Full Confounder Model";

%mvgee(mydataset=master_mv_outreach, myoutcome=sexwork_outreach_l6m, myvars=social_cohes_score age lgbq2s_any

race housing_l6m serve_where_r police_harass_l6m unsafesex_any_l6m ninj_l6m, myrefvars=lgbq2s_any(ref='0: No')

race(ref='0: White') housing_l6m(ref='0: No') serve_where_r(ref='1: Outdoor/public space') police_harass_l6m(ref='0: No')

unsafesex_any_l6m(ref='0: No') ninj_l6m(ref='0: No'), mysubject=aesha_code, mycorr=exch);

ods rtf startpage = now;

ods proclabel = "5.0: Most Parsimonious Confounder Model, outcome is sexwork_outreach_l6m";

title1 j=left font='Arial' color=black bold "Most Parsimonious Confounder Model";

%mvgee(mydataset=master_mv_outreach, myoutcome=sexwork_outreach_l6m, myvars=social_cohes_score

race housing_l6m serve_where_r police_harass_l6m ninj_l6m, myrefvars=lgbq2s_any(ref='0: No')

race(ref='0: White') housing_l6m(ref='0: No') serve_where_r(ref='1: Outdoor/public space') police_harass_l6m(ref='0: No')

unsafesex_any_l6m(ref='0: No') ninj_l6m(ref='0: No'), mysubject=aesha_code, mycorr=exch);

ods rtf close;
